# Proceedings of the Oregon State Dental Society

**Published:** 1876-01

**Authors:** J. R. Cardwell, G. W. Gray


### Proceedings of Societies.

### PROCEEDINGS OF THE OREGON STATE DENTAL SOCIETY.

The Oregon State Dental Society met persuant to adjournment, in the hall of Albany Engine Co., and in the absence of the President, was called to order by the Vice-President, Dr. J. R. Cardwell.

The roll being called the following members answered to their names: Drs. J. R. Cardwell, G. H. Chance, G. W. Gray, J. H. Hatch, G. W. Meredith, T. L. Nicklin, E. O. Smith, L. S. Skiff and J. Welch.

Minutes of previous meeting read and approved.

On motion, a committee was appointed to report upon candidates for membership, consisting of Drs. Skiff, Chance and Gray.

The application of Dr. S. J. Barber was reported upon favorably, and upon ballot was unanimously elected.

An interesting communication from the President, Dr. Wm. F. Thompson (now residing in San Francisco,) was read and on motion, placed on file.

Reading of essays being in order, Dr. G. W. Gray presented one on "Irregularities of the teeth and the best means of correcting the same."

The subject of the paper was then taken up and generally discussed, all the members participating.

On motion, a committee, consisting of Drs. Nicklin, Smith and Welch was appointed to revise the Constitution and By-Laws of this Society, and report during this session. On motion, adjourned to meet at the office of Dr. G. W. Gray, at 7½ p. m.

### EVENING SESSION.

Society met pursuant to adjournment, Dr. J. R. Cardwell in the Chair.

Minutes of previous meeting were read and approved.

Dr. J. Welch, Treasurer of the Society, made his report, which was referred to the Committee on Finance, consisting of Drs. Skiff, Meredith and Chance, who, after examination, reported the accounts kept correctly, when, on motion, the report of the Treasurer was adopted and ordered placed on file.

Reading of essays being in order, Dr. J. H. Hatch presented an interesting paper on "Operative Dentistry."

Dr. J. R. Cardwell then read an able production on "Neuralgia."

Discussion being in order, the subject of capping and filling teeth over exposed nerves was taken up and discussed at considerable length, in which all the members participated, giving various methods of treatment.

Dr. G. H. Chance made a motion that the Theses, as read by Drs. Gray, Hatch and Cardwell, be forwarded to the dental journals for publication. The motion was carried.

On motion, the Society adjourned, to meet at the hall of Albany Engine Co., to-morrow, Thursday, at 8 o'clock, a. m.

### MORNING SESSION.

Society met at 8 a. m., Dr. J. R. Cardwell in the Chair.

Members present as on yesterday, with the addition of Dr. Rubell, of Monmouth.

Minutes of previous session read and approved.

A general discussion was then held on the subject of the various materials used for filling teeth. Gold, silver, tin, amalgam, Wood's metal, Os-artificial, Hill's stopping, etc., were discussed; and while it was agreed that each substance may have some advantage in peculiar cases, yet gold was decided to be superior to all other materials in purity and durability.

On motion, adjourned, to meet at the office of Dr. Gray at 2 p. m., to witness his operating in clinic at that time.

AFTERNOON SESSION.

The Society met at the office of Dr. Gray at 2 p. m., when Dr. Gray proceeded to illustrate his mode of operating, by filling a large posterior approximal cavity over an exposed pulp in the left superior second bicuspid. The operation was skillfully performed, to the satisfaction of the Society.

On motion, adjourned to 7½ p. m.

EVENING SESSION.

Society met at Dr. Gray's office at 7½ p. m.

Letters were read from Dr. Kochler, of Portland, and Dr. Miller, of Eugene, regreting their inability to attend the session of the Society. On motion, they were excused.

Dr. Chance presented the following paper in reference to the length of time necessary to prepare students for the practice of our profession:

Believing that the time has arrived when uniformity should govern the dental profession with regard to the education of those who are to come after us; and fully realizing that no man can become a skillful dentist, alike an honor to his profession and a benefit to community, unless he has received a thorough dental education, therefore,

*Resolved*, That we, the undersigned members of the Dental Profession of the State of Oregon, mutually pledge ourselves not to take a student for a less term than three years, and that we will use all legitimate means to induce our students, before assuming practice on their own responsibility, to attend a full course in some reputable Dental College, either during their pupilage or as soon after as practicable.

Signed; J. H. Hatch, Geo. H. Chance, Geo. W. Gray, J. R. Cardwell, T. L. Nicklin, W. H. Rubell, John Welsh, J. W. Meredith, Q. S. Skiff, E. O. Smith.

The Society then proceeded to discuss the merits of the various materials as a base for artificial dentures; also, extracting, etc. Time being limited the discussions were cut short without reaching any definite conclusions.

The following officers were elected for the ensuing year.

President—Dr. J. R. Cardwell, Portland.

Vice-President—Dr. G. W. Gray, Albany.

Rec. Sec.—Dr. G. H. Chance, Portland.

Cor. Sec.—Dr. J. H. Hatch, Portland.

Treasurer—Dr. Q. S. Skiff, Salem.

Executive Committee—Drs. T. I. Nicklin, J. H. Hatch and Geo. H. Chance.

The Committee on Revision reported, and the report was adopted.

Dr. Hatch presented and illustrated an ingenious and novel method of making corundum disks with which all were highly gratified.

A remarkable specimen of Japanese dentistry was presented by Dr. Hatch. A set of teeth made of actual ivory inserted in a base carved out of smooth hard wood. The teeth are inserted with a wooden dowel. The place of the molars is supplied by some rough iron tacks driven in the wooden base. They exhibit a good deal of patient whittling but are clumsy and awkward; yet they would be a great help to an aristocratic "John" in masticating his rice.

On motion, the thanks of the Society were unanimously tendered to Albany Engine Co., No. 1. for their courtesy in granting us the free use of their hall. Also to Drs. Gray and Smith for their hospitality during our visit to Albany.

On motion, adjourned to meet at Portland on the last Wednesday of June, 1876, at 1 p. m.

J. R. Cardwell, Pres. G. W. Gray, Sec.

